# Prevalence of Psychological Distress and Associated Factors in Nursing Students during the COVID-19 Pandemic: A Cross-Sectional Study

**DOI:** 10.3390/ijerph181910358

**Published:** 2021-10-01

**Authors:** Fumiya Tanji, Yuki Kodama

**Affiliations:** 1 Faculty of Nursing, Japanese Red Cross Akita College of Nursing, Akita 010-1490, Japan; 2 Faculty of Nursing, Tokyo University of Information Sciences, Chiba 265-8501, Japan; y3kodama21@rsch.tuis.ac.jp

**Keywords:** psychological distress, resilience, insomnia, nursing student, COVID-19

## Abstract

Little is known about psychological distress and its associated factors during the COVID-19 pandemic among nursing students, especially during the initial spread. The present study aimed to investigate the prevalence of psychological distress among Japanese nursing students after the first semester of 2020 with shifted classes and practice online. Furthermore, we examined whether factors such as health issues and resilience were associated with psychological distress. The data were obtained from a cross-sectional, self-administered questionnaire survey at a Japanese nursing college from September to November 2020 (*n* = 289). We collected data such as sociodemographic variables, medical history, insomnia, and resilience (Bidimensional Resilience Scale). Psychological distress was measured using the K6 scale (scores ≥ 5). The prevalence of psychological distress was 58.5%. The multivariate Poisson regression analysis found a negative association between innate resilience factors, and positive associations between medical history, insomnia, and psychological distress. The present study showed that more than half of the study participants experienced high levels of psychological distress. Our results suggest the importance of monitoring mental health among nursing students, focusing not only on health issues, but also personality components such as innate resilience during the prolonged COVID-19 pandemic.

## 1. Introduction

The coronavirus disease 2019 (COVID-19) pandemic first emerged in Wuhan, China, in December 2019, and it has since spread worldwide. As of 23 August 2021, there were more than 200 million confirmed cases and 4 million deaths worldwide [1], with approximately 1.3 million confirmed cases and 1500 deaths in Japan [2]. The COVID-19 outbreak forced countries around the world to initiate lockdowns or partially regulate behavior and has impacted educational settings, which have shifted from face-to-face classes to online classes [3]. These changes in educational methods have placed a burden on university students in developing infrastructure, acquiring digital skills, and feeling lonely due to thinning interpersonal relationships [4]. Therefore, university students have also experienced stress due to the sudden change in educational methods, in addition to the COVID-19 infection itself.

Among university students, those in fields such as nursing and medicine often face significant stress throughout their education [5,6]. These factors include extensive curricula, numerous academic requirements, and difficult examinations and assignments [5,6]. Before the COVID-19 pandemic emerged, a systematic review reported that the prevalence of psychological distress, such as depression and depressive symptoms, was 27.2% among medical students [7]. Recent studies soon reported the prevalence of moderate or severe psychological distress measured by the Kessler Psychological Distress Scale (K6) in the months after the COVID-19 pandemic emerged: 26.6% among Chinese medical science students [8] and 28.5% among Japanese medical students [9]. When comparing the prevalence of psychological stress preceding the pandemic and following it, there seems to be little difference. However, another study reported that the prevalence of moderate or severe psychological distress measured by K6 was 19.7% among university freshmen of various disciplines following the pandemic [10]. Thus, the prevalence of psychological distress may be higher among students in the medical science field than among non-medical science students. Fushimi reported that the number of students attending mental health consultations increased in the latter part of 2020 [11], which implies that the prevalence of psychological stress may have increased. However, there are few reports on the prevalence of psychological stress among medical science students several months after the continued changes in educational methods due to the COVID-19 pandemic. The present study conducted a web-based survey among nursing students in the second semester of 2020, after they had taken classes and practiced online in the previous semester.

Previous studies have shown related factors, such as health issues (e.g., chronic disease as a COVID-19 infection risk, and insomnia) and socioeconomic status (SES), for psychological distress following the COVID-19 pandemic [12,13]. In addition, when a social stress event such as COVID-19 occurs, an individual’s personality influences their mental health. For example, the impact of the 1997–1998 economic crisis on the completed suicide risk differed according to personality subscales; suicide risk increased among those with higher neuroticism after the economic crisis, whereas there was no such association for other personality subscales [14]. This is due to the ability of a specific personality type to cope with stress and hardship.

The ability of an individual to bounce back or recover from stress is known as resilience [15]. There are many scales to measure resilience, but the Bidimensional Resilience Scale (BRS) developed by Hirano et al. classifies it into two dimensions: innate and acquired factors [16]. This scale was constructed using the Temperament and Character Inventory (TCI) scale developed by Cloninger et al. as an external criterion [17]. They divided personality into two dimensions: temperament, which is independently heritable, and character, which is acquired. Innate resilience factors of the BRS are strongly related to temperament, and the acquired resilience factors of the BRS are related to character [16]. It is well known that resilience is associated with psychological distress [18,19]. To our knowledge, however, no study has examined whether this association involves innate or acquired resilience.

The present study aimed to investigate the prevalence of psychological distress and the factors associated with psychological distress among Japanese nursing students after taking classes and practicing online in the first semester of 2020. We included the learning environment, health issues, and resilience as associated factors.

## 2. Materials and Methods

### 2.1. Study Design, Participants, and Data

We conducted a cross-sectional, web-based, self-administered questionnaire survey at a nursing college in Akita, in northern Japan. Akita Prefecture in Japan is located in a suburban area and the number of people infected with COVID-19 is low compared to urban areas such as Tokyo. We conducted the survey anonymously from 25 September to 30 November 2020. During this period, the study participants were required to take hybrid classes (online and face-to-face), could not practice at hospitals but instead online or on campus, and had to limit unnecessary outings in their daily lives. We distributed the questionnaire to all undergraduate students regardless of year (*n* = 430), and a total of 293 students (68.1%) participated in the survey. We excluded four respondents who had no psychological distress data. Thus, 289 participants were included in the analysis.

After obtaining written consent from the participants, we collected and used the data from the anonymous survey. This study was reviewed and approved by the Ethics Committee of the Japanese Red Cross Akita College of Nursing (approval number: 2020–113).

### 2.2. Use of Secondary Data

To estimate the prevalence of psychological distress before the COVID-19 pandemic among people around the same age as the study participants (aged 20–24 years), we used aggregated data from the Comprehensive Survey of Living Conditions (CSLC) in 2019 as secondary data [20]. The CSLC has been conducted annually through small-scale and large-scale surveys every three years by the Ministry of Health, Labor, and Welfare of Japan [21]. The large-scale survey included a health questionnaire, with questions covering psychological distress. The number of participants aged 20–24 years in the 2019 CSLC was 4595, and 4481 participants responded to questions about psychological distress [20]. We estimated the prevalence of psychological distress before the COVID-19 pandemic using their data.

### 2.3. Assessments

#### 2.3.1. Sociodemographic Data

Sociodemographic data were collected on the year in college, sex, medical history (presence or absence), residence type (living alone, or living with family or in university housing), type of entrance examination (recommendation or written exam), and cumulative grade point average (GPA) as a measure of academic performance.

Academic performance was measured using GPA for fourth-year students and a functional grade point average (f-GPA) for first-to-third-year students, calculated using the raw score (0–100), grade point (GP), and credits, where GP = (raw score–55)/10. The f-GPA is calculated as Σ (GP × credit)/Σ credit and ranges from 0 to 4.5 (continuous variable). We classified the participants into three groups based on academic performance-specific tertiles: lower, middle, and higher.

#### 2.3.2. Psychosocial Factors

We collected data on resilience, insomnia, concerns in college life (keeping up with classes, getting a job and proceeding to the next level of education, and friendship in college), subjective economic status (severe, normal, or affluent), and sense of fulfilment in college life (fulfilling or not fulfilling).

Resilience measured using the Bidimensional Resilience Scale (BRS) confirmed the validity and reliability of the data [16], which has been used in many studies [22,23]. The BRS comprises 21 items in two dimensions: 12 items for innate factors and 9 items for acquired factors. Each of the 21 items was assessed using three questions with five response levels, ranging from 1 (strongly disagree) to 5 (strongly agree). Thus, the total point scores of innate and acquired resilience factors ranged from 12 to 60, and from 9 to 45, with higher scores indicating a greater impact for each factor. For each factor, we classified the participants into three groups according to tertiles: lower (12–39 points), middle (40–45 points), and higher groups (46–60 points) for innate factors; and lower (9–31 points), middle (32–35 points), and higher groups (36–45 points) for acquired factors.

Insomnia was measured using the Athens Insomnia Scale (AIS) [24,25]. The total score ranges from 0 to 24. We classified participants with scores of ≥6 as having insomnia and those with scores ≤ 5 as not having insomnia [24].

Concerns in college life were assessed by asking the question, ‘Do you have any concerns in your college life related to the following items: (1) keeping up with classes, (2) whether to get a job or to proceed to the next level of education as desired, or (3) friendship in college?’ The participants were asked to choose one of the following four answers: ‘many, some, few’, and ‘none’. For the above three items, we classified participants into two groups: presence (many or some) and absence (few or none).

#### 2.3.3. Psychological Distress

Psychological distress was measured using the Kessler Psychological Distress Scale (K6) [26]. The Japanese version of K6 has been validated previously [27]. Total point scores ranged from 0 to 24, with a higher score indicating severe psychological distress. In accordance with previous studies [28,29], we classified participants into three groups using cut-off points of 4/5 and 12/13: low (0–4 points), moderate (5–12 points), and severe (13–24 points).

### 2.4. Statistical Analysis

For baseline characteristics, we evaluated the proportion of each measurement and the mean (standard deviation (SD)) of each BRS score. We estimated the prevalence of psychological distress (low, moderate, and severe) among the current study participants and the 2019 CSLC participants’ secondary data, then conducted a chi-squared test.

We assessed factors associated with psychological distress (K6 score ≥ 5) during the COVID-19 pandemic. First, multiple imputations were conducted for missing values of medical history, academic performance, living arrangement, insomnia, and BRS (acquired resilience factors) to create 10 output datasets. For these imputations, we used all variables as predictor variables. After the multiple imputation, we conducted a univariate Poisson regression analysis with a robust error variance to estimate the prevalence ratios (PRs) and 95% confidence intervals (CIs) using the following factors: medical history, residence type, type of entrance examination, GPA, innate resilience factor, acquired resilience factor, insomnia, concerns in college life (keeping up with classes, getting a job and proceeding to the next level of education, and friendship in college), subjective economic status, and sense of fulfilment with college life. Then, we simultaneously included variables for year at college and sex and performed a multivariate analysis including all factors, with *p* < 0.10 identified in the univariate analysis. We also conducted a sensitivity analysis using a K6 score of ≥13 as the dependent variable.

All statistical analyses were performed using IBM SPSS Statistics version 25 (IBM SPSS Software Group, Chicago, IL, USA). All statistical tests were two-sided analyses, and differences were considered significant at *p* < 0.05.

## 3. Results

Table 1 shows the characteristics of all participants. The number of participants in each college year for the first, second, third, and fourth years was 85 (29.4%), 85 (29.4%), 58 (20.1%), and 61 (21.1%), respectively. The proportion of women was 91.7% (*n* = 265). Among the participants who had concerns in college life, 139 (48.1%) were concerned about keeping up with classes, 112 (73.4%) were concerned about getting a job and proceeding to the next level of education, and 75 (26.0%) were concerned about friendship in college. The number of participants with insomnia (AIS score ≥ 6 points) was 105 (36.3%). The mean (SD) BRS score for all participants was 41.97 (7.90) for the innate resilience factors and 32.90 (4.92) for the acquired resilience factors.

Table 2 compares the prevalence of psychological distress among study participants during the COVID-19 pandemic with that of Japanese people around the same age (aged 20–24 years) before the pandemic. The prevalence of psychological distress among study participants during the pandemic was 41.5% low, 46.0% moderate, and 12.5% severe. On the other hand, the distribution of psychological distress among Japanese people of the same age prior to the pandemic was 68.6% (low), 25.1% (moderate), and 6.3% (severe). Compared to the findings before the pandemic, the prevalence of psychological distress in this study’s participants was approximately doubled in both the moderate and severe levels. The results of the chi-square test showed a significant difference in the prevalence of psychological distress during and before the COVID-19 pandemic (*p* < 0.001).

Table 3 shows the results of the univariate Poisson regression analysis with robust error variance after multiple imputations. The independent variables that showed significant positive associations with psychological distress (K6 score ≥ 5) were medical history, concerns in college life (keeping up with classes, getting a job and proceeding to the next level of education, and friendship in college), subjective economic status, sense of fulfilment in college life, and insomnia (*p* < 0.05). The independent variables that showed a significant negative association with psychological distress were innate and acquired resilience factors (*p* < 0.001).

Table 4 shows the results of the multivariate Poisson regression analysis with robust error variance after multiple imputations. Psychological distress (K6 score ≥ 5) was used as the dependent variable, and factors with *p* < 0.10 in Table 3 were used as independent variables. There were significant positive associations in medical history (PR = 1.30; 95% CI, 1.07–1.59; *p* = 0.009) and insomnia (PR = 1.26; 95% CI, 1.06–1.50; *p* = 0.008). In terms of resilience, a significant negative association was found only in the highest group for the innate resilience factor score (PR = 0.70; 95% CI, 0.53–0.92; *p* = 0.011), while there was no association between the acquired resilience factors and psychological distress.

We conducted a sensitivity analysis using a K6 score of ≥13 as the dependent variable (data not shown). There was a significant positive association between insomnia and psychological distress (PR = 2.42; 95% CI, 1.29–4.55; *p* = 0.007). Although medical history and innate resilience factors showed similar associations with psychological distress, as shown in Table 4, there was no significant association. In contrast to the results in Table 4, there was a significant positive association between concerns about friendship in college and psychological distress (PR = 2.03; 95% CI, 1.13–3.66; *p* = 0.019).

## 4. Discussion

In the present study, the prevalence of moderate and severe psychological distress among Japanese nursing students during the COVID-19 pandemic was 58.5% (46% for moderate and 12.5% for severe). This prevalence was approximately twice as high (both moderate and severe psychological distress) as that of the similarly aged Japanese participants in the 2019 CSLC secondary data. The present study showed that medical history, insomnia, and innate resilience factors were associated with psychological distress among Japanese nursing students during the COVID-19 pandemic. The results of the sensitivity analysis showed the same trends.

In previous studies of medical science students before the COVID-19 pandemic, the prevalence of depression or depressive symptoms had been reported to be 32.6% among Taiwanese nursing students and 27.2% among medical students worldwide [7,30]. In addition, previous studies reported that the prevalence of psychological distress a few months after the COVID-19 pandemic was 28.5% among Japanese medical students and 26.6% among Chinese medical science students [8,9]. These two studies assessed psychological distress (scores ≥5 points) using the same K6 scale as in the present study. The present study, which was conducted after study participants had taken classes and practiced online in the first semester of 2020, showed approximately twice the prevalence of psychological distress (58.5%) as the above two studies. This result suggests that prolonged changes in the learning environment and behavioral restrictions may have worsened psychological distress as well as increased its prevalence. Therefore, it is important to monitor the mental health of medical science students, including those in nursing, during prolonged stressful events such as COVID-19.

The present study showed an association between health issues such as chronic diseases, insomnia, and psychological distress during the COVID-19 pandemic. Patients with COVID-19 are more likely to have chronic disease, and those with chronic disease are more likely to die due to COVID-19 [31,32]. Younger people had longer media exposure and greater access to COVID-19 information, and those with a medical history were more likely to feel vulnerable to COVID-19, which might be associated with higher psychological distress [13,33]. The present study showed a strong association between insomnia and psychological distress in both the main and sensitivity analyses. Among the study participants, there were many comments that the shift from face-to-face classes and practice to online coursework resulted in an increase in assignments and a decrease in sleep time. In corroboration, Marelli et al. reported that the prevalence of insomnia increased among university students due to the introduction and continuation of online classes during the COVID-19 pandemic [12]. These changes in the learning environment may have influenced the association between insomnia and psychological distress. It might be important to screen and monitor students with health issues early on. In addition, it might also be necessary to improve and build upon current online education methods to reduce the burden on students.

In the present study, participants with higher innate resilience factors were significantly associated with lower psychological distress, whereas there was no association between acquired resilience factors and psychological distress. Among medical students, self-efficacy and self-esteem predicted psychological distress in the months after the COVID-19 pandemic emerged [9]. Self-efficacy and self-esteem are known to promote resilience [34], which means that the resilience here is acquired. Another study reported that the association between COVID-19-related stressful experiences and acute stress disorder was mediated by resilience among university students [35], although resilience was not divided into innate and acquired factors. The acquired trait of resilience may affect how university students cope with acute stress immediately after a stressful event. Isobe et al. reported that only innate resilience factors were positively associated with self-rated health, and self-rated health was negatively associated with psychological distress among dental hygienists and dental hygiene students [22]. This previous study suggests that it is beneficial to focus on heritable traits of innate resilience factors, such as optimism and sociability, in order to maintain self-rated health status. Therefore, innate resilience factors may play a role in coping with stressful events, not only immediately after them, but also when stressful events are prolonged. Our results suggest that it may be important to focus on students’ innate resilience (temperament) to maintain their mental health.

This study has some limitations. First, we had no data for specific responses to changes in the learning environment and types of chronic illnesses. Second, we had no primary data on resilience and psychological distress among study participants before the COVID-19 pandemic emerged, because we conducted a cross-sectional study at a single point in time after the pandemic. Psychological distress caused by the COVID-19 pandemic may have influenced resilience and insomnia (i.e., reverse causality). Third, this study was conducted at a single site. Not only between countries, but also within Japan, there would be differences in the COVID-19 infection rates among different regions and in the changes in educational methods across different universities. The study area is located in a suburban area, and the number of people infected with COVID-19 here is low compared to urban areas. However, the occurrence of just a few cases can easily lead to discrimination and hate, which may have made the people of the suburban area more sensitive to fears and anxiety of COVID-19 infection. The generalizability of our findings to Japanese nursing students may be limited.

## 5. Conclusions

The present study showed that the prevalence of psychological distress among Japanese nursing students was approximately twice as high as that among Japanese people of the same age prior to the COVID-19 pandemic. In addition, it was revealed that psychological distress may be affected by innate resilience factors, as well as health issues such as insomnia and chronic diseases. Our results suggest the importance of continuously monitoring mental health among nursing students during a prolonged COVID-19 pandemic. Further studies should prospectively explore the prevalence of psychological distress and its related factors.

## Figures and Tables

**Table 1:** Characteristics of the study participants (*n* = 289).

Characteristics			*n* (%)	Mean (SD ^1^)
Year in college				
	First		85 (29.4)	
	Second		85 (29.4)	
	Third		58 (20.1)	
	Fourth		61 (21.1)	
Sex				
	Male		24 (8.3)	
	Female		265 (91.7)	
Medical history				
	Presence		85 (37.1)	
	Absence		64 (27.9)	
	Missing		10 (4.4)	
Type of entrance examination				
	Recommendation		23 (10.0)	
	Written exam		204 (89.1)	
Academic performance				
	Higher		92 (31.8)	
	Middle		96 (33.2)	
	Lower		94 (32.5)	
	Missing		7 (2.4)	
Living arrangement				
	Living alone		94 (32.5)	
	Living with family or in university housing		194 (67.2)	
	Missing		1 (0.3)	
Concerns in college life				
	Keeping up with classes			
		Presence	139 (48.1)	
		Absence	150 (51.9)	
	Getting a job and proceeding to the next level of education			
		Presence	112 (73.4)	
		Absence	77 (26.7)	
	Friendship in college			
		Presence	75 (26.0)	
		Absence	214 (74.0)	
Subjective economic status				
	Normal or affluent		174 (60.2)	
	Severe		115 (39.8)	
Sense of fulfilment with college life				
	Fulfilling		241 (83.4)	
	Not fulfilling		48 (16.6)	
Insomnia ^2^				
	Presence		105 (36.3)	
	Absence		183 (63.4)	
	Missing		1 (0.3)	
Resilience (BRS ^3^)				
	Innate resilience factors			41.97 (7.90)
	Acquired resilience factors ^4^			32.90 (4.92)

^1^ SD = standard deviation. ^2^ Participants with Athens Insomnia Scale scores of ≥6 were defined as having insomnia. ^3^ BRS = Bidimensional Resilience Scale. ^4^ There were 2 missing data on the acquired resilience factors.

**Table 2:** Comparison of psychological distress levels between study participants and the 2019 CSLC participants (*n* = 289).

		Psychological Distress (K6 ^2^ Score)			
		Low (0–4)	Moderate (5–12)	Severe (13–24)	*p*
During the COVID-19 pandemic					
	Study participants (%)	120 (41.5)	133 (46.0)	36 (12.5)	<0.001
Pre-pandemic					
	2019 CSLC ^1^ participants aged 20–24 years (%)	3075 (68.6)	1123 (25.1)	283 (6.3)	

^1^ CSLC = Comprehensive Survey of Living Conditions, ^2^ K6 = Kessler 6-Item Psychological Distress Scale.

**Table 3:** Univariate Poisson regression analysis after multiple imputation (*n* = 289).

		PR ^3^	95% CI ^2^		*p*
Medical history		1.48	1.22	1.78	<0.001
Type of entrance examination		0.87	0.72	1.06	0.176
Academic performance		1.04	0.92	1.17	0.566
Living alone		0.97	0.78	1.19	0.751
Concerns in college life					
	Keeping up with classes	1.42	1.16	1.73	0.001
	Getting a job and proceeding to the next level of education	1.34	1.03	1.74	0.027
	Friendship in college	1.45	1.21	1.74	<0.001
Subjective economic status		1.30	1.07	1.57	0.007
Sense of fulfilment in the college life		1.41	1.16	1.71	<0.001
Insomnia		1.42	1.18	1.72	<0.001
Resilience (BRS ^1^)					
	Innate resilience factors	0.97	0.96	0.98	<0.001
	Acquired resilience factors	0.97	0.95	0.98	<0.001

^1^ BRS = Bidimensional Resilience Scale, ^2^ CI = confidence interval, ^3^ PR = prevalence ratio.

**Table 4:** Multivariate regression analysis after multiple imputation (*n* = 289).

		PR ^2^	95% CI ^1^		*p*
Year in college		1.03	0.94	1.13	0.512
Female		0.92	0.70	1.21	0.543
Medical history		1.30	1.07	1.59	0.009
Concerns in college life					
	Keeping up with classes	1.21	0.995	1.47	0.057
	Getting a job and proceeding to the next level of education	1.25	0.96	1.62	0.100
	Friendship in college	1.15	0.97	1.37	0.113
Subjective economic status		1.10	0.92	1.32	0.287
Sense of fulfilment in the college life		1.18	0.98	1.42	0.074
Insomnia		1.26	1.06	1.50	0.008
Innate resilience factors score (tertile)					
	Middle (40–45 points)	0.82	0.65	1.03	0.082
	Higher (≥46 points)	0.70	0.53	0.92	0.011
Acquired resilience factors score (tertile)					
	Middle (32–35 points)	0.88	0.70	1.11	0.294
	Higher (≥35 points)	0.90	0.70	1.15	0.401

^1^ CI = confidence interval, ^2^ PR = prevalence ratio.

## Data Availability

The data presented in this study are available upon request from the corresponding author. The data are not publicly available because of privacy concerns.
